# Online Holocaust and Genocide Education in Undergraduate Nursing: A Mixed-Methods Evaluation of Ethical Integrity and Professional Identity

**DOI:** 10.3390/nursrep16030096

**Published:** 2026-03-10

**Authors:** Anat Romem, Zvika Orr

**Affiliations:** 1 Henrietta Szold School of Nursing, Faculty of Medicine, Hadassah, The Hebrew University of Jerusalem, Kalman Y. Man Street, Jerusalem 91120, Israel; 2 Herzog Medical Center, 96 Givat Shaul Street, Jerusalem 9103702, Israel; 3 Selma Jelinek School of Nursing, Jerusalem College of Technology, 11 Beit Hadfus Street, Jerusalem 9548311, Israel; orr@g.jct.ac.il; 4 CERMES3—Centre for Research on Medicine, Science, Health, Mental Health, and Society, CNRS—INSERM—EHESS—Université Paris Cité, 7 Guy Môquet Street, 94801 Villejuif, France

**Keywords:** nursing education, professional identity, ethical integrity, Holocaust education, genocide studies, patient advocacy, reflective learning, online learning

## Abstract

**Background:** Professional identity and ethical integrity are foundational to nursing practice and are shaped in part by educational experiences. This study evaluated an online Holocaust and genocide educational seminar delivered to fourth-year Bachelor of Science in Nursing (BSN) students and explored how students linked seminar content to professional identity formation, ethical vigilance, and patient advocacy. **Methods:** We conducted a descriptive mixed-methods educational evaluation. Students completed an anonymous pre-seminar survey (demographics, motivations for studying nursing, self-identified desirable professional qualities, and self-rated knowledge of the Holocaust and other genocides) and an anonymous post-seminar feedback survey with four open-ended questions. Quantitative items were summarized descriptively; qualitative data were analyzed using inductive qualitative content analysis. **Results:** Of the 205 students who attended the seminar, 133 completed the pre-seminar survey, and 110 completed the post-seminar survey. Students reported high baseline knowledge of the Holocaust but limited knowledge of the Armenian and Rwandan genocides. The five themes that emerged are as follows: (1) ethical judgment and the influence of nurses; (2) patient advocacy and social justice; (3) the effect of historical and contemporary trauma on students’ learning experience; (4) genocide awareness and prevention; and (5) approaches to education and content presentation. **Conclusions:** Carefully facilitated Holocaust and genocide education, delivered through interactive online pedagogy and structured debriefing, may support late-stage nursing students’ reflection on ethical integrity and professional identity during the transition to professional practice.

## 1. Introduction

Professional identity and ethical integrity are foundational components of nursing practice, shaping how nurses interact with patients, respond to ethical dilemmas, and view their roles within the healthcare system [1,2,3,4,5,6]. These concepts intersect in different ways and are affected by nursing education. Lessons learned from the history of nursing can be particularly relevant and effective [7,8,9,10,11]. This study examines students’ perceptions of the influence of Holocaust and genocide education on the development of professional identity and ethical integrity among fourth-year nursing students who participated in an educational seminar on the Holocaust and genocide.

### 1.1. Professional Identity in Nursing

Professional identity in nursing is a dynamic construct that emerges from a nurse’s personal understanding of themselves, their roles, and their clinical experiences. Professional identity explores the internalization of values, norms, and characteristics of the nursing discipline, allowing nurses to think, act, and feel aligned with their professional roles [1,2,12]. This identity formation provides a foundation for how nurses perceive and conduct their practice [2,3,4,5].

Professional identity is shaped by adherence to ethical standards and codes of practice. The Nursing and Midwifery Council outlines the ethical standards that nurses must uphold, emphasizing the importance of integrity, respect, and compassion in patient care [13]. These standards provide a framework for professional behavior, guiding nurses in their daily practice and ensuring they maintain ethical integrity [13,14]. Role modeling is essential in the formation of professional identity [15]. Role models significantly influence the development of a nurse’s professional identity by demonstrating ethical behavior, empathy, and patient advocacy. Exposure to positive role models can enhance a nurse’s commitment to ethical integrity and social justice [15].

Professional identity also plays a key role in nurses’ decisions to remain in the profession, as they can align their personal and professional values [16,17,18]. This alignment is crucial for maintaining ethical integrity and advocating for patients effectively [16,17,18]. A strong professional identity, built on ethical principles and historical awareness, enables nurses to navigate complex ethical dilemmas and advocate for social justice within the healthcare system [16,17,19,20].

### 1.2. Ethical Integrity in Nursing

Ethical integrity in nursing represents a persistent commitment to upholding moral and professional principles in every facet of practice. It is anchored in the core values of the profession, as delineated by the American Nurses Association’s Code of Ethics, which serves as a foundational guide for ethical decisions and professional interactions [21]. Demonstrating ethical integrity involves practicing honesty, respecting individuals, and adhering to principles of autonomy and justice while maintaining a focus on beneficence, such as actively doing good, and non-maleficence, such as avoiding harm in patient care.

Ethical integrity obliges nurses to advocate for patients’ rights, ensuring respect for their autonomy and dignity, even in difficult circumstances [14,22,23]. Similarly, beneficence and non-maleficence remain central to ethical integrity, requiring nurses to prioritize patient welfare and avoid harm, even in challenging ethical contexts [24].

Beyond individual practice, ethical integrity allows the cultivation of ethical environments within healthcare organizations. Provision 6 of the Code of Ethics emphasizes the collective responsibility of nurses to establish and sustain ethical work environments that support safe, high-quality care [21]. This involves not only individual accountability but also collaboration with interdisciplinary teams, advocating for fairness in treatment, and engaging in continuous learning to enhance ethical practice [25].

Ethical integrity and professional identity are cultivated through education and reflective practice. This enables nurses to maintain honesty and humility while respecting patient beliefs and providing equitable care [26]. Integrating professional identity development into nursing curricula also helps students internalize ethical principles and develop a strong sense of professional responsibility. This approach prepares nursing students to face ethical dilemmas confidently and maintain their commitment to patient advocacy and social justice [27,28].

Ethical vigilance refers to an ongoing attentiveness and critical awareness that enables individuals to recognize subtle ethical shifts and question practices that may gradually undermine humane standards of care. In this study, ethical vigilance is used as a modificatory ethical stance regarding early warning signs of dehumanization, boundary erosion, propaganda, and moral normalization of harm rather than as a synonym for ethical integrity or professional identity.

#### The Relationship Between Professional Identity and Ethical Integrity

Although closely related, professional identity and ethical integrity are not interchangeable. Professional identity refers to the developing sense of self through which individuals internalize the norms, values, role expectations, and behaviors of nursing, ultimately learning to think and act as a nurse [1,2,3,4,5,6]. Ethical integrity, by contrast, refers to consistent ethical principles and professional standards in decisions and behaviors, particularly under pressure, ambiguity, or institutional constraint [21,22,23,24,25]. In this framing, professional identity provides the internal compass (who I am as a nurse and what nursing stands for), while ethical integrity is the behavioral and decision-level expression of that compass (what I do when values are tested). Therefore, these two constructs overlap; both concern values and moral responsibility, yet they remain analytically distinct: a learner can articulate identity commitments without consistently translating them into ethical action, and conversely, they can comply with rules without deep identity internalization. This distinction guided our interpretation of the themes below, including how students described role responsibility and belonging as identity-salient reflections versus ethical vigilance, advocacy, and speaking up as ethical integrity-salient reflections.

### 1.3. Holocaust and Genocide Education in Nursing

Holocaust and genocide studies among nursing students provide an example of how education and reflective practice can cultivate ethical integrity and professional identity. Studies have found that incorporating these lessons into nursing school curricula encourages students to confront discrimination and structural racism in the healthcare system [29,30].

Holocaust education emphasizes the perspectives of victims and survivors and increases understanding and compassion among the nurses who treat them [31]. Thus, understanding historical atrocities equips nurses with deeper ethical and critical awareness and a commitment to preventing inhumane practices in healthcare [8,9,10,11,32]. This education also encourages nurses to be vigilant against unethical behavior and to advocate for patient rights, becoming critical intellectuals who integrate lessons from Holocaust and genocide studies into practice [8,29]. A study conducted in Australia found that such education not only honors the memory of victims but also instills a sense of professional integrity and ethical responsibility in nursing students [30].

Despite growing interest in Holocaust and genocide education in nursing, there remains limited empirical evidence on how online Holocaust and genocide educational interventions function as professional formation experiences, particularly at the transition to the practice stage, linking historical trauma education to the development of professional identity and ethical integrity in nursing students. In addition, little is known about how students integrate familiar collective memory, such as the Holocaust, with less familiar genocide cases and how this integration interacts with contemporary sociopolitical stressors that may amplify emotional salience. Addressing these gaps is important for designing ethically responsible, trauma-informed pedagogy that strengthens ethical vigilance and advocacy without unintended harm.

### 1.4. Educational Intervention: Seminar Design and Delivery

In this study, Holocaust and genocide educational seminars were integrated into the final semester of a Bachelor of Science in Nursing (BSN) program, representing a strategically timed and impactful educational intervention. The curriculum was delivered on four separate seminar days across two weeks to different classes, with each attending one full seminar day lasting eight hours. For each class, a pre-seminar survey was distributed before the scheduled seminar day, and a post-seminar feedback survey was distributed immediately following completion of that class’s seminar day. This structure ensured that pre-seminar responses reflected baseline perceptions and knowledge for each class before exposure to the seminar content, and post-seminar responses captured immediate reflections following the learning experience.

We intentionally placed the seminar in the final semester since professional identity consolidation and ethical decision-making are especially salient during the transition-to-practice period. Capstone pedagogy typically emphasizes applied experiences; however, transition is also a period when students confront moral complexity, power dynamics, and accountability in real clinical settings and benefit from structured meaning that connects prior learning to professional responsibility. In this design, the seminar functioned as a short, intensive integrative component that complements applied capstone work, rather than substituting for it, by explicitly linking historical lessons to advocacy, moral vigilance, and professional accountability in contemporary practice. The transition-to-practice and capstone literature supports senior-level integrative learning experiences that bridge theory to role enactment and strengthen readiness for professional practice [1,2,3,4,5,6]. This focus supports students in internalizing the lessons of historical atrocities, cultivating a professional identity that prioritizes ethical vigilance and advocacy in practice [10,30,32].

The seminar format allows for active learning through discussion, reflection, and engagement with survivor testimonies, historical accounts, and ethical case studies. In particular, Holocaust education emphasizes the lived experiences of victims and survivors, prompting nursing students to critically examine their roles as advocates for vulnerable populations [31]. This educational approach aims to inspire a commitment to ethical nursing practice, ensuring that the lessons of history are preserved and applied to prevent future injustices [30,32].

The seminar was structured in two sessions and delivered via the Zoom platform (version 6.0), enabling synchronous learning with interactive, multimedia resources. The first session concentrated on Holocaust studies and included several topics. It opened with a screening of the film “The House” [33], followed by a discussion about the story of ghetto partisan units and underground resistance. A joint study session and discussion was conducted after students viewed the “Deadly Medicine: Creating the Master Race” exhibition by the United States Holocaust Memorial Museum in Washington, D.C., online. Next, students participated in an interactive tour of the virtual “Camps Hall” exhibition of the Ghetto Fighters’ House. Finally, the seminar concluded with an innovative online workshop, “Voices in the Void: The Rescue of Danish Jews”, which focused on the moral and social implications of this unique rescue story.

The second session explored the history and course of genocide through the Armenian and Rwandan genocides. It included the following topics: definition and motives for genocide, mass violence in the modern world, Gregory Stanton’s [34] ten stages of genocide and genocide subset, moral inversion, and prediction of genocide. Given the constraints of a one-day seminar, genocide education was not intended as comprehensive coverage of all genocides. Instead, the curriculum used selected cases as Holocaust studies alongside the Armenian and Rwandan genocides as pedagogic examples to (i) contrast contexts and mechanisms across time and geography, (ii) apply a genocide-process framework (such as Stanton’s model) to concrete historical material, and (iii) address observed student knowledge gaps beyond the Holocaust.

Following each content block, a dedicated 60 min facilitated debriefing was conducted in the Zoom environment. Debriefing was guided by structured reminders, such as emotional reactions, ethical tensions identified, relevance to nursing practice, and boundaries or uncertainties raised by the material, with explicit norms supporting psychological safety, including permission to pause and step away, respectful dialog, and validation of distress as a normal response to traumatic material. Students were also reminded of support pathways available through the academic institution and encouraged to seek support if the content elicited significant distress. This design was particularly important because multiple students explicitly linked learning to contemporary events and described heightened emotional difficulty.

### 1.5. Study Aims

This study aimed to evaluate a Holocaust and genocide educational seminar delivered to fourth-year nursing students by (1) describing students’ self-identified professional qualities and motivations for choosing nursing; (2) describing students’ baseline self-rated knowledge of the Holocaust and other genocides; and (3) through post-seminar written reflections, exploring how students perceived links between the seminar content and their developing professional identity, ethical integrity, patient advocacy, and social responsibility.

We conceptualized this educational evaluation using Kirkpatrick’s four-level model, focusing on outcomes appropriate to the study design and data sources. In this iteration, our evaluation primarily addresses Level 1 (Reaction) through immediate post-seminar reflections (emotional responses, perceived relevance, perceived appropriateness, and timing) and Level 2 (Learning) through descriptive pre-seminar self-rated knowledge and post-seminar perceived connections between historical content and nursing practice. The study does not assess Level 3 (Behavior) or Level 4 (Results), and therefore, claims are limited to students’ self-reported perceptions and immediate learning reactions [35].

## 2. Materials and Methods

We conducted a descriptive, mixed-methods educational evaluation. Quantitative data were collected using an anonymous pre-seminar survey assessing demographics, motivations for choosing nursing, desirable professional qualities, and baseline self-rated knowledge of the Holocaust and other genocides.

Qualitative data were collected using an anonymous post-seminar feedback survey consisting of four open-ended questions, eliciting emotional responses, perceived relevance to practice, reflections on the contemporary context, and perceived similarities and differences between the Holocaust and other genocide cases.

### 2.1. Ethics

The study was approved by the Ethics Committee of the Jerusalem College of Technology (approval number: 006_24). All data were collected anonymously to ensure the protection of student privacy. No personally identifiable information was recorded. The data were securely stored in encrypted files accessible only to the research team. Furthermore, all data handling procedures complied with the Jerusalem College of Technology Ethics Committee’s data protection and privacy regulations to safeguard student confidentiality. Given that participants were students, we considered potential power differentials and the risk of perceived coercion. Participation in both surveys was voluntary, conducted via an anonymous web-based form with an explicit consent item; selecting non-consent automatically ended the survey, and no responses were saved. Participation or non-participation did not influence course standing or evaluation. To reduce demand characteristics, surveys were anonymous, and responses were analyzed in aggregate; pre- and post-responses were not linkable at the individual level.

Given the emotionally intense nature of Holocaust and genocide content, students were provided with structured debriefing time following each session and were reminded of available institutional support services if they experienced distress. Students were also informed they could stop participating in the research component at any time without any consequences.

We recognize that researchers’ positionality may shape topic selection, facilitation, and interpretation. To reduce expectancy effects and social desirability pressures, participation was voluntary and anonymous, and analyses were presented as descriptive accounts of students’ self-reported perceptions rather than objective measures of competence or behavior. We also explicitly considered the contemporary context referenced by students and treated politically charged terminology in quotations, reflecting a participant’s intent rather than study conclusions.

### 2.2. Study Procedure

Before the seminar, students were invited to complete an anonymous web-based questionnaire assessing demographics, motivations for choosing nursing, self-identified desirable professional qualities, and self-rated knowledge of the Holocaust and other genocides.

Immediately following the seminar day, students were invited to complete an anonymous post-seminar feedback survey consisting of four open-ended questions designed to elicit emotional responses, perceived relevance to clinical practice, reflections on the contemporary context, and perceived similarities and differences between the Holocaust and other genocides.

### 2.3. Pre-Seminar Survey

The pre-seminar survey was designed to gather data from nursing students ahead of a seminar, focusing on their motivations for choosing nursing, nurses’ professional qualities they value, their own desired qualities as future healthcare professionals, and their knowledge of past genocides (Table 1). Items were designed to align with seminar objectives, prior nursing ethics, and the professional identity literature; however, formal psychometric validation was not conducted for this study-specific instrument. Therefore, quantitative findings are reported descriptively and interpreted as exploratory baseline self-reports.

### 2.4. Post-Seminar Survey

The post-seminar survey was intentionally designed as an immediate qualitative reflection instrument to capture emotionally salient learning, perceived relevance to practice, and contextual meaning rather than as a repeated measures survey of the pre-seminar constructs. Items were developed by the research team to align with the seminar’s learning objectives and were informed by the relevant literature on Holocaust and genocide education and professional identity formation in nursing. The self-rated “knowledge” item captured students perceived familiarity and understanding of these topics rather than serving as an objective test of factual knowledge. Given that the data were collected anonymously and could not be linked at the individual level, we did not quantify within-person change from pre- to post-seminar.

The post-seminar survey had four open-ended questions to collect the students’ feedback about the seminar and its topics:

1. Please share your thoughts and feelings during and after the study day.
2. How do you see the connection between the content you learned on the study day and clinical work? What insights, knowledge, or approach would you like to implement as a nurse in the health system (if any)?
3. During and after the study day, did you think about the current situation? If so, what did you think about? What is the relevance of the content learned to our times?
4. How do you see the similarities and differences between the Holocaust and other genocide cases? What is similar and different between the thoughts and feelings that arose in you during the study of these two topics?

### 2.5. Data Analysis

Quantitative items from the pre-seminar survey were analyzed descriptively (frequencies, percentages, means, and standard deviations as appropriate) to summarize student characteristics, motivations for choosing nursing, self-identified desirable professional qualities, and baseline self-rated knowledge of the Holocaust and other genocides. Qualitative data from the four post-seminar open-ended questions were analyzed using inductive qualitative content analysis, progressing from codes to categories and then to themes.

Responses were read repeatedly for familiarization, coded inductively to capture meaningful units of content, and then organized into candidate themes. Themes were reviewed and refined to ensure internal coherence and clear distinctions between themes, and representative excerpts were selected to illustrate each theme. Two authors independently coded an initial subset of responses, discussed coding differences to refine the codebook, and then coded the full dataset, meeting iteratively to consolidate categories and agree on final themes [36]. The findings are presented as the students’ perceptions and reflections following the seminar.

## 3. Results

### 3.1. Demographic Characteristics

Out of the 205 students who participated in the Holocaust and genocide seminar, 133 responded to the pre-seminar questionnaire (a response rate of 65%). Of these participants, 88.2% were female. The average age was 26.2 years (SD = 6.85 years). All respondents were Jewish. In terms of religious affiliation, the majority identified as religious (n = 72, 54.1%) or ultra-Orthodox (n = 58, 43.6%).

### 3.2. Motivation for Studying Nursing

Students were asked to give their primary reason for choosing to study nursing. The most prevalent reason was “to be there for the patients” (47.3%), followed by “to achieve academic and professional self-fulfillment” (35.9%). None selected “to help and provide quality of life care” (Table 2).

### 3.3. Perceived Qualities of Nurses

Table 3 provides an overview of the qualities students value in an ideal nurse who would be caring for their parents compared with the qualities they wish to embody as professionals. The most desired quality for an ideal nurse caring for their parents was professionalism (54.1%), followed by kindness (46.6%) and a holistic approach to all types of patients (39.1%). Notably, less emphasized traits were sociability (0.8%) and optimism (1.5%). The most commonly identified qualities the students aim to embody as professionals include professionalism (44.7%), honesty and reliability (43.2%), and a holistic approach to all types of patients (40.9%).

### 3.4. Pre-Seminar Knowledge of Genocides

In the pre-seminar survey, most students reported extensive knowledge of the Holocaust (83.5%) but minimal knowledge of the Rwandan genocide (78.2%). Over 47% reported intermediate knowledge of the Armenian genocide. The degree of knowledge for each genocide is detailed in Table 4.

The demographic characteristics of the participants, as well as their motivations for studying nursing and perceived qualities of nurses, significantly influenced their engagement with the seminar themes. Their extensive knowledge of the Holocaust, compared with limited awareness of other genocides, shaped their reflections on ethical integrity, patient advocacy, and historical trauma.

### 3.5. Inductive Qualitative Content Analysis

Out of the potential 205 respondents, 110 participated in the seminar feedback evaluation, achieving a response rate of 54%. Five main themes were identified in the content analysis of the feedback. These include ethical judgment and the influence of nurses, patient advocacy and social justice, the effect of historical and contemporary trauma on students’ learning experience, genocide awareness and prevention, and approaches to education and content presentation. These themes reflect the broader topics discussed during the seminar.

#### 3.5.1. Theme 1: Ethical Judgment and the Influence of Nurses

There was a broad consensus among the students that reflecting on ethical judgment is necessary in nursing. This lack of reflection was evident during the Holocaust and other genocides. One student remarked,

*“It scared me how an entire nation can be convinced that what they are doing is right-this can also apply to my work as a nurse.”*

The seminar highlighted the significance of values in nursing. It is not enough to know the technical aspects of nursing; there needs to be an inherent understanding of the values and morals steeped in nursing. One participant noted,

*“I think I am more aware of the enormous caution needed in the face of moral dilemmas.”*

The awareness of one’s influence as a nurse was also acknowledged, especially in relation to how it may affect one’s practice. The students emphasized the responsibility of nurses and other medical professionals in light of this influence, and how they failed during the Holocaust.

*“The first lecture made me think about how propaganda and education can influence not only the choices people make but even the justification of actions that contradict all morality.”*

*“Medical professionals have tremendous power, and during the Holocaust, they used this power to kill patients or people with disabilities instead of helping them and showing compassion.”*

#### 3.5.2. Theme 2: Patient Advocacy and Social Justice

Advocacy was a strong component of the discussions. Students shared that they felt an obligation to stand by their patients, no matter who they are, and even if they disagreed with their choices.

*“As a nurse, I will always be there for others, even if our opinions differ, their pain is my pain as well!”*

*“In the healthcare system, it is important to provide treatment and response to every patient, regardless of who they are.”*

The commitment to oppose injustice was evident in the reflections. Students stated their belief in incorporating social justice into their practice. Some students also emphasized the importance of speaking up for patients.

*“By incorporating a critical lens on privilege, power dynamics, and systemic injustices into my practice, I hope to contribute to positive change in the healthcare system.”*

*“I hope to apply the idea that even as a nurse, if we see immoral and inappropriate things, not to remain silent.”*

#### 3.5.3. Theme 3: The Effect of Historical and Contemporary Trauma on Students’ Learning Experience

The inductive qualitative content analysis revealed that the impact of historical trauma, particularly of the Holocaust, is deeply felt among the students. The students also discussed how learning about the Holocaust and genocide was very difficult in the wake of the atrocity of 7 October 2023.

*“It was very difficult because it brought me back to October 7th and all the horrible images and videos from there... very sad!”*

*“It was hard for me to discuss genocide when we are in the midst of a war for our existence as a Jewish people in our land.”*

*“I also think it was not entirely appropriate to give such a lecture during such a period.”*

The use of memory as a tool for education and preventing future atrocities was a key aspect to applying the historical lessons to practice. The students expressed that, as a collective, they have responsibility to make sure these atrocities do not occur again.

*“It is our responsibility to learn from history and ensure that such genocides do not occur again.”*

*“It is important to remember the lessons of the Holocaust to prevent it.”*

#### 3.5.4. Theme 4: Genocide Awareness and Prevention

Students frequently linked the seminar content to contemporary events and used the seminar framework to interpret perceived threats and moral challenges. Some students explicitly used the term “genocide” when describing current events, while others focused on ethical boundaries, risks of moral simplification, and the emotional difficulty of engaging with genocide-related content during an ongoing national crisis. To avoid misinterpretation, we emphasize that the participants’ view of “genocide” in relation to contemporary events reflects their personal perceptions and moral language during an ongoing crisis. This study does not seek to adjudicate legal or political classifications of contemporary conflicts; rather, these quotations are retained to accurately represent the contextual meaning that shaped students’ learning experience.

*“I thought about what happened on October 7th and the horrifying reality we are in today.”*

*“The current reality is very painful and surreal that exactly what happened is happening again even though not many years have passed since the Holocaust!”*

*“I think the current reality of what happened on October 7th is a genocide.”*

In this framework, some of the students shared that people make cynical use of compassion when discussing contemporary atrocities. They stressed that it is important for nurses to also recognize boundaries.

*“Although it is moral to be for the weak, there are people who exploit this compassion and claim to cover up their crimes with their suffering.”*

#### 3.5.5. Theme 5: Approaches to Education and Content Presentation

The educational approach and content delivery received mixed reactions from students. This feedback is important to better adjust delivery channels to students. The engagement with content was generally high, with students finding the lessons on Nazi propaganda and the lecturer’s sensitivity valuable. The use of educational materials played a significant role in the learning experience. These included multimedia aids, such as videos, exhibits, and objects. Students shared that these helped them better understand the seminar material.

*“The exhibition was very interesting; I really love historical things that actually existed during the period being told.”*

*“I really learned about the propaganda the Nazis used with the help of technology.”*

However, transitioning from these initial reactions, the educational sessions introduced more complex and challenging material that elicited a different set of responses. Some participants were confronted with new and troubling information that expanded their understanding of historical atrocities.

*“I didn’t know about all the history of genocide and how many times it has occurred in various countries.”*

*“The part of the lecture about the Holocaust was fascinating, later it became quite analytical and a bit heavy; in any case, the concept of eugenics was new to me.”*

*“It was a tough day but enlightening, to understand that doctors were the ones who took the first steps towards extermination, all under the guise of reason and progress, where is the line in our generation, there are many questions that come from this.”*

*“That seminar was a bit difficult; I felt that it came with an approach that isn’t entirely how I see or believe in things. On the other hand, the medical and nursing side that was in Germany at the beginning of the Holocaust was renewed for me. Many things in the first lecture were not simple, but it is important to confront them, and in the end, my great-grandmother was murdered by the Nazis because she was hospitalized in a psychiatric hospital, so I also felt that I was encountering this side in me, with my story.”*

## 4. Discussion

This descriptive mixed-methods evaluation examined fourth-year nursing students’ baseline knowledge of the Holocaust and other genocides; their stated motivations and desired professional qualities; and their post-seminar reflections on ethical integrity, professional identity formation, and patient advocacy. Overall, students’ written responses suggest that Holocaust and genocide education can function as a structured catalyst for reflection on moral responsibility, the professional power of healthcare workers, and the importance of resisting dehumanization in clinical and organizational contexts.

Across themes, students’ reflections pointed to both professional identity formation and ethical integrity, but in different ways. Identity-salient reflections emphasized nurses’ role, power, responsibility, and self-positioning as future professionals: “what kind of nurse I want to be,” professional pride, and alignment with nursing’s moral purpose. Ethical integrity-salient reflections emphasized moral vigilance and action commitments: recognizing propaganda and dehumanization as ethical risk factors, naming duties of advocacy, and describing thresholds for speaking up when witnessing injustice or unethical practice. The overlap between these two constructs was most visible when students connected historical lessons to present-day bedside conduct, where identity meaning (“this is who we are as nurses”) was articulated as a rationale for integrity-based action: “therefore we must advocate, resist dehumanization, and protect dignity”.

Understanding the processes that occurred in the Holocaust and other genocides enables nurses to cultivate a deeper appreciation of the ethical dimensions of their profession. This educational strategy is designed to deepen nurses’ awareness of the moral implications of their work, thereby equipping them to manage the complexities of patient care in an ethically conscientious manner.

### 4.1. Motivations and Aspirations in Nursing

Of the motivations that drive students to choose the nursing profession, the quantitative data point to a predominant focus on patient-centered care and personal growth, emphasizing the multidimensional nature of nursing, including clinical skills, emotional intelligence, and ethical considerations. The most frequently cited reason for pursuing nursing was “To be there for the patients” (47.3%), reflecting the central roles of empathy, compassion, and direct patient interaction in shaping students’ career aspirations. This aligns with broader professional standards in nursing, which prioritize patient advocacy and the delivery of holistic care [37]. The prominence of this response suggests that many students are drawn to nursing out of a deep-seated desire to provide meaningful support during vulnerable times in patients’ lives [19].

The second most prevalent reason, “To achieve academic and professional self-fulfillment” (35.9%), highlights the profession’s appeal as a pathway for academic and personal development. This response indicates that many students view nursing not only as a means to serve others but also as a career that offers opportunities for growth, continuous learning, and professional advancement. This dual focus on altruism and self-improvement reflects the dynamic nature of nursing, which requires a balance of technical expertise, emotional resilience, and adaptability.

Interestingly, none of the participants selected “To help and provide quality of life care” as their primary motivation. This absence may indicate a gap in students’ understanding of how nursing contributes to quality of life, or it could reflect a broader cultural or educational framing of the nursing profession that emphasizes acute care over quality-of-life interventions [20,38]. This finding warrants further exploration, as promoting quality of life is a foundational component of nursing practice, particularly in geriatric, palliative, and chronic care settings.

The strong emphasis on patient-centered care and professional fulfillment suggests that nursing curricula should continue to emphasize the development of clinical and interpersonal skills while fostering opportunities for self-reflection and career planning [39]. At the same time, the low prioritization of motivations related to advancing medical science or ensuring quality of life care highlights potential areas for greater emphasis in nursing education.

When examining the qualities students associate with nursing excellence, numerous key aspects emerge from both student reflections and survey data. These findings highlight the multifaceted nature of nursing, where technical expertise, interpersonal attributes, and emotional intelligence converge to meet patient and familial care needs effectively.

Nursing students prioritize professionalism (54.1%), kindness (46.6%), and a holistic approach to all types of patients (39.1%) for the ideal nurse they would choose to care for their parents. These preferences reflect an expectation of competence, compassion, and comprehensive care, which align with foundational principles of nursing practice. The emphasis on honesty and reliability (36.1%) and patience (30.1%) indicates the importance of trust and consistent care delivery, particularly in scenarios involving vulnerable populations, such as aging parents.

Interestingly, qualities like sociability (0.8%) and optimism (1.5%) received minimal emphasis, suggesting that while these traits may enhance interpersonal interactions, they are considered secondary to core professional attributes. This finding may point to a preference for tangible, measurable qualities over more abstract or personality-driven traits in the context of caregiving.

A parallel yet slightly different prioritization is evident when nursing students reflect on the qualities they wish to embody in their professional roles. The most-cited attributes, including professionalism (44.7%), honesty and reliability (43.2%), and a holistic approach to all types of patients (40.9%), mirror those valued in their chosen nurses. The inclusion of kindness (28.8%), empathy (28%), and patience (27.3%) highlights a strong emphasis on emotional and interpersonal skills in their self-perception as future nurses.

Lower-ranked qualities, such as optimism (2.3%) and sense of humor (0.8%), suggest that these traits, while potentially beneficial, are not perceived as central to professional identity. Instead, students appear to focus on qualities that directly impact patient outcomes and foster trust in clinical settings.

The observed alignment between the qualities students attribute to an ideal nurse and those they personally aspire to embody suggests that students recognize a responsibility to uphold, in their own practice, the same professional standards they expect of others. The qualities of kindness and empathy are valued differently when it comes to caring for parents versus a personal professional evaluation. Kindness is particularly emphasized in parental care, where it is valued at 46.6% compared with 28.8% in self-assessment by professionals. This distinction arises because kindness offers immediate reassurance to family members observing the care of their loved ones, making it easily noticeable and appreciated. Its visible impact quickly assures families of the quality of care while also establishing a nurturing environment crucial for the well-being of patients, making it a top priority in this setting.

The overlap between the attributes students endorse for ideal nurses and those they claim as personal aspirations indicates an internalization of professional norms, with students positioning themselves as accountable to the same standards they apply to others. Conversely, within their professional self-assessments, nurses often place a higher value on clinical skills and professional behavior over kindness. They view kindness as foundational but not the sole focus, emphasizing the need to balance interpersonal skills with the technical competencies necessary for effective practice. 

Unlike kindness, empathy was more prominent among the qualities students desired for themselves than among those they attributed to an ideal nurse caring for their parents. The effects of empathy are not as immediately visible, making it a refined aspect of patient care. Families expect empathy as a standard nursing quality, but it may not be explicitly recognized unless it significantly impacts care outcomes. In professional self-reflection, empathy is highly valued due to its deep impact on enhancing patient communication and trust.

The seminar feedback and qualitative content analysis revealed five themes that, while distinct, are interwoven and collectively emphasize the importance of a socially conscious, ethically grounded, and historically informed nursing practice [26]. The seminar’s discussions encouraged students to think critically about the broader societal and historical contexts in which they will practice and to consider the deep impact that nurses can have on both individual patients and society at large [20].

One of the seminar’s key outcomes was fostering reflection on the ethical judgment and influence of nurses (Theme 1). Students perceived the importance of internalizing values such as honesty, respect, and autonomy, aligning their personal principles with the broader moral framework of nursing practice. The historical context of the Holocaust served as a moving reminder of the power and influence of healthcare professionals, emphasizing the necessity for nurses to act with integrity and accountability [29].

The seminar stressed the critical role of ethical integrity in nursing, particularly in advocating for patient rights and social justice (Theme 2). Students articulated their commitment to standing by patients, regardless of personal differences, and ensuring equitable care. This aligns with ethical principles such as beneficence and non-maleficence, which prioritize patient welfare and the avoidance of harm [24]. Many participants expressed a heightened awareness of systemic injustices in healthcare, emphasizing the need to integrate a critical view of privilege and power dynamics into their practice.

The seminar also explored the effect of historical and contemporary trauma on students’ learning experience (Theme 3). Students identified connections between historical atrocities and contemporary events, deepening their understanding of the importance of memory as a tool for ethical reflection and prevention. The emotional impact of learning about the Holocaust was apparent, particularly in the wake of the events of 7 October 2023.

Additionally, the seminar provided a framework for students to engage with contemporary issues of genocide and human rights violations (Theme 4). Many students reported connections between the Holocaust and modern atrocities, reflecting on the importance of ethical vigilance in preventing inhumane practices. However, some participants experienced the emotional and contextual challenges of discussing genocide during a time of national crisis. While these reflections revealed the difficulties of engaging with sensitive topics, they also highlighted the importance of fostering resilience and ethical clarity in future nurses [14,40].

### 4.2. Educational Approaches and Student Engagement

From the perspective of innovative education, a key contribution of this study is the demonstration of a scalable, technology-enabled pedagogy that combines synchronous online delivery with curated virtual museum exhibitions, multimedia resources, and structured debriefing. Such approaches may help nursing programs address curriculum pressures while preserving depth of learning and facilitating ethical reflection in geographically and culturally diverse cohorts [10,39,41,42].

The seminar’s educational approach played a significant role in identifying the connection between professional identity and ethical integrity. Interactive materials engaged students and enhanced their understanding of the seminar’s content. The sensitivity of the lecturer and the focus on Nazi propaganda were particularly impactful for many participants. However, the timing of the seminar presented emotional challenges for some students, highlighting the need to consider contextual factors when delivering content. Despite these challenges, students described the seminar as meaningful and reported intentions to apply insights related to ethical awareness, moral boundaries, and patient advocacy in future practice.

Recent evidence supports deliberately designed educational strategies to strengthen ethical sensitivity and moral reasoning in nursing students, commonly through case-based discussion, simulation, and structured reflection [43,44]. Given that nursing students may experience moral distress during training and early transition to practice, explicitly teaching moral resilience and ethical coping strategies may further reinforce the type of ethical vigilance and advocacy emphasized in this seminar [27,45].

Given that Holocaust and genocide education can evoke strong emotional responses, equity-centered trauma-informed pedagogy is particularly relevant; it emphasizes psychological safety, transparent facilitation, and relational accountability when learners engage with distressing material [46]. In parallel, emerging work on neurodivergent nursing students emphasizes the importance of inclusive learning design and flexible placement supports to promote belonging and retention [47,48].

Finally, the priorities highlighted in including artificial intelligence will increasingly intersect with ethical integrity and professionalism [49]. Recent reviews emphasize both educational opportunities and the need for explicit ethical guidance regarding bias, privacy, transparency, and academic integrity when AI tools are introduced into nursing curricula [49,50,51].

### 4.3. Implications for Curriculum and Trauma-Informed Facilitation

For nursing curricula, these findings support positioning Holocaust and genocide education as an advanced, professionally integrative learning experience near transition to practice, explicitly tied to ethics, advocacy, and professional identity formation rather than treated as general history content. To strengthen safety and learning, delivery should use a trauma-informed facilitation frame: advance preparation and clear learning goals; transparent signaling of emotionally intense segments; structured debriefing with norms for respectful dialog; and clear pathways to support for students who experience distress. Trauma-informed educational practices in nursing emphasize psychological safety, trust, and relational accountability, elements that are especially important when learners engage with atrocities, moral injury, and contemporary parallels [2,14,45,46]. Attention to psychological safety is not only protective but also improves reflective depth and the likelihood that students can translate emotionally evocative material into professional commitments, such as ethical vigilance, advocacy, and responsibility.

### 4.4. Limitations

Surveys were collected anonymously; therefore, pre- and post-seminar responses could not be linked at the individual level, which prevents estimation of within-person change over time. Accordingly, the mixed-methods design should be interpreted as descriptive and exploratory: it integrates baseline self-reports with post-seminar reflections to characterize perceived learning and meaning rather than to test change attributable to the intervention.

Furthermore, the qualitative component was designed to support depth and contextual understanding rather than statistical generalization. Thus, contextual specificity is treated as a feature of the qualitative findings, which are presented as situated insights that complement the descriptive survey results.

Interpretation is also shaped by the broader context in which the seminar occurred. The seminar and evaluation took place during a period of collective stress and trauma, and several participants referenced contemporary events in their reflections (including 7 October 2023). This context may have heightened emotional salience, shaped interpretive framing, and influenced students’ language when discussing mass violence. Some participants used the term “genocide” to describe current events, whereas others questioned the timing of engaging genocide-related content during an ongoing crisis and emphasized the emotional difficulty of doing so. These statements are reported as participants’ perspectives; the study did not aim to determine contemporary political or legal classifications. Given that the reflections are closely tied to the sociopolitical moment in which they were produced, transferability to cohorts learning the same content under more stable conditions may be limited.

Additional limitations should be noted. First, both the survey and written reflections rely on self-report and may be influenced by social desirability or perceived expectations when responding to morally charged material; therefore, the findings should be interpreted as perceived learning and anticipated professional implications rather than verified behavioral change. Second, post-seminar participation was voluntary, raising the possibility of non-response bias if students who were less engaged or more distressed were less likely to submit feedback. Finally, the contemporaneous context of national trauma and war may have intensified emotional responses and shaped meaning, further reinforcing that the findings are context-bound and exploratory rather than causal estimates of intervention effects.

## 5. Conclusions

In this cohort of fourth-year nursing students, Holocaust and genocide education was associated with rich reflections on professional identity, ethical vigilance, patient advocacy, and social responsibility. Students linked historical content to contemporary ethical challenges and to the potential influence of nurses within healthcare systems.

These findings support the educational value of integrating carefully facilitated Holocaust and genocide learning activities, alongside structured debriefing, within undergraduate nursing curricula, particularly near the transition to professional practice. By implementing these strategies, nursing education can develop professionals who excel in clinical care while upholding ethical integrity, advocating for patients, and promoting social justice.

### Future Research

Future research should strengthen causal inference and clarify mechanisms of impact in three ways. First, studies should use linked longitudinal designs (pre-seminar, post-seminar, and follow-up into early clinical practice) to assess within-person change in professional identity formation and ethical integrity. This work should pair validated quantitative instruments with reflective qualitative data to capture both measurable trajectories and the meaning processes that accompany them. Second, comparative designs are needed to identify which pedagogical components drive outcomes. Examples include comparing the seminar with alternative ethics curricula, evaluating synchronous versus blended delivery formats, and systematically varying debriefing structures to test their contribution to learning, integration, and retention. Third, research should directly assess facilitator preparation and the quality of trauma-informed delivery. Measures of psychological safety, pacing, containment, and debriefing processes should be examined as moderators that may shape both educational gains and distress-related outcomes.

These priorities are particularly important because immersion in historical trauma can be emotionally burdensome and may shape learning in non-linear ways. This complexity may be amplified when students are simultaneously living through contemporary collective stressors, which can heighten vulnerability and influence interpretive framing. Accordingly, future studies should evaluate not only potential educational benefits but also potential adverse effects, including heightened distress, moral polarization, and disengagement. In parallel, investigators should test whether trauma-informed facilitation and structured debriefing mitigate emotional burden while supporting reflective learning and professional growth.

## Figures and Tables

**Table 1:** The structure of the pre-seminar survey.

Motivations for Choosing to Study Nursing	Students were asked to reflect on their personal motivations for studying nursing.
Desirable Qualities in Nurses	Participants were asked to rate three qualities they would want to see among nurses caring for their parents.
Personal Qualities as Professionals	Students were asked to reflect on the three qualities they believe are most important for themselves as future healthcare professionals.
Knowledge of Genocides	A series of questions was used to assess participants’ knowledge of significant historical events, such as the Holocaust and the Armenian Genocide, on a Likert scale of 1 (“I don’t know anything”) to 5 (“I know a lot”).
Demographic Information	Sex, age, and religious affiliation (secular, traditional, religious, or ultra-Orthodox).

**Table 2:** The reasons for choosing the nursing profession (n = 131; participants could select only one answer).

Reason for Choosing to Study Nursing	n (%)
To be there for the patients	62 (47.3%)
To achieve academic and professional self-fulfillment	47 (35.9%)
To find what I love in the field	12 (9.1%)
To promote a healthy lifestyle	6 (4.6%)
To develop a varied and independent career	2 (1.5%)
To advance medical science and technology	1 (0.8%)
To obtain a high salary and job security	1 (0.8%)
To help and provide quality of life care	0 (0%)
Total	100%

**Table 3:** Comparison of qualities desired in an ideal nurse and oneself as a professional (n = 133, caring for parents; n = 132, as a professional).

Quality	Nurse Caring for Parents % (n)	Oneself as a Professional % (n)
Professionalism	54.1% (72)	44.7% (59)
Kindness	46.6% (62)	28.8% (38)
A holistic approach to all types of patients	39.1% (52)	40.9% (54)
Honesty and reliability	36.1% (48)	43.2% (57)
Patience	30.1% (40)	27.3% (36)
Human qualities	24.8% (33)	26.5% (35)
Empathy	20.3% (27)	28% (37)
Advanced knowledge	10.5% (14)	12.9% (17)
Emotional support skills	9% (12)	10.6% (14)
Emotional stability	7.5% (10)	7.6% (10)
Assertiveness	6.8% (9)	15.9% (21)
Trust and acceptance	4.5% (6)	3% (4)
Ambition	3.8% (5)	5.3% (7)
Thoroughness	3% (4)	3.8% (5)
Decisiveness	3% (4)	7.6% (10)
Intelligence	3% (4)	3.8% (5)
Academic and high-level knowledge	3% (4)	6.8% (9)
Optimism	1.5% (2)	2.3% (3)
Sociability	0.8% (1)	1.5% (2)
Sense of humor	—	0.8% (1)

**Table 4:** Knowledge related to genocide and the Holocaust (n = 133).

Degree of Knowledge	Armenian Genocide n (%)	Rwanda Genocide n (%)	Holocaust n (%)
Extensive Knowledge	8 (6%)	2 (1.5%)	111 (83.5%)
Intermediate Knowledge	63 (47.4%)	27 (20.3%)	5 (3.7%)
Limited Knowledge	62 (46.6%)	104 (78.2%)	17 (12.8%)
Total	100%	100%	100%

## Data Availability

De-identified data may be made available from the corresponding author upon reasonable request, subject to institutional approvals and privacy requirements.

## Public Involvement Statement

Patients and the public were not involved in the design, conduct, reporting, or dissemination plans of this educational evaluation.

## Guidelines and Standards Statement

This study is reported in accordance with the Good Reporting of a Mixed Methods Study (GRAMMS) framework and the Strengthening the Reporting of Observational Studies in Epidemiology (STROBE) guidance, as applicable.

## References

[1] American Association of Colleges of Nursing (AACN) (2019). AACN’s Vision for Academic Nursing. https://www.aacnnursing.org/News-Information/Position-Statements-White-Papers/Vision-for-Nursing-Education.

[2] American Association of Colleges of Nursing (AACN) (2021). The Essentials: Core Competencies for Professional Nursing Education. https://www.aacnnursing.org/Portals/0/PDFs/Publications/Essentials-2021.pdf.

[3] Güner Y., Turhal E., Üçüncüoğlu M., Tuncel B., Akturan S., Keleş Ş. (2021). The formation of professional identity in nursing. Turk. J. Bioeth..

[4] Mao A., Lu S.E., Lin Y., He M. (2021). A scoping review on the influencing factors and development process of professional identity among nursing students and nurses. J. Prof. Nurs..

[5] Mbalinda S.N., Najjuma J.N., Gonzaga A.M., Livingstone K., Musoke D. (2024). Understanding and barriers of professional identity formation among current students and recent graduates in nursing and midwifery in low resource settings in two universities: A qualitative study. BMC Nurs..

[6] Benner P., Sutphen M., Leonard V., Day L. (2010). Educating Nurses: A Call for Radical Transformation.

[7] Hall E.O.C., Joensen A.L., Malchau Dietz S. (2023). Historiographic and biographic accounts of Faroese nurses’ training and health-promoting work from 1910 to the end of the 1930s. Int. J. Circumpolar Health.

[8] Ben-Sefer E. (2006). Lessons from the past for contemporary Australian nursing students: The Nazi euthanasia program. Nurse Educ. Pract..

[9] Kruse J.A., Wald H.S. (2025). Legacy of medicine and nursing during the Holocaust and its contemporary relevance: Addressing implicit and explicit bias and health care inequities. J. Contin. Educ. Nurs..

[10] Shields L., Manning J., McAllister M., O’Brien D., Shields K., Emeto T.I., Brien D.L., Murray J., Benedict S. (2022). Nursing ethics and the Holocaust: Pilot of an innovation in teaching. Holocaust Stud..

[11] Ben-Sefer E., Sharon D., Benedict S., Shields L. (2014). Using the “Euthanasia” programs to teach nursing ethics. Nurses and Midwives in Nazi Germany: The “Euthanasia Programs”.

[12] Ghorbani A., Mohammadi N., Rooddehghan Z., Bakhshi F., Nasrabadi A.N. (2023). Transformational leadership in development of transformative education in nursing: A qualitative study. BMC Nurs..

[13] Nursing and Midwifery Council (2018). The Code: Professional Standards of Practice and Behaviour for Nurses, Midwives and Nursing Associates. https://www.nmc.org.uk/standards/code/.

[14] Rushton C.H. (2016). Moral resilience: A capacity for navigating moral distress in critical care. AACN Adv. Crit. Care.

[15] Koh E.Y.H., Koh K.K., Renganathan Y., Krishna L. (2023). Role modelling in professional identity formation: A systematic scoping review. BMC Med. Educ..

[16] Wald H.S. (2015). Professional identity (trans)formation in medical education: Reflection, relationship, resilience. Acad. Med..

[17] Kruse J.A., Wald H.S. (2023). Legacy of the role of medicine and nursing in the Holocaust: An educational intervention to support nursing student professional identity formation and ethical conduct. Nurs. Educ. Perspect..

[18] Kristoffersen M. (2021). Does professional identity play a critical role in the choice to remain in the nursing profession?. Nurs. Open.

[19] Vabo G., Slettebø Å., Fossum M. (2022). Nursing students’ professional identity development: An integrative review. Nord. J. Nurs. Res..

[20] Vila V., Zhuang J., Tan E., Thorne S. (2018). Reflections on nursing educational advancement within diverse and evolving national cultural contexts. Int. J. Nurs. Educ. Scholarsh..

[21] American Nurses Association Code of Ethics for Nurses. https://codeofethics.ana.org/home.

[22] Epstein B., Turner M. (2015). The nursing code of ethics: Its value, its history. Online J. Issues Nurs..

[23] Varkey B. (2021). Principles of clinical ethics and their application to practice. Med. Princ. Pract..

[24] Tiruneh M.A., Ayele B.T. (2018). Practice of code of ethics and associated factors among medical doctors in Addis Ababa, Ethiopia. PLoS ONE.

[25] Trobec I., Starcic A.I. (2015). Developing nursing ethical competences online versus in the traditional classroom. Nurs. Ethics.

[26] Sandvik A.H., Hilli Y. (2023). Understanding and formation—A process of becoming a nurse. Nurs. Philos..

[27] Bulfone G., Bressan V., Zerilli I., Vinci A., Mazzotta R., Ingravalle F., Maurici M. (2024). Moral distress and its determinants among nursing students in an Italian university: A cross-sectional study. Nurs. Rep..

[28] Rasmussen P., Henderson A., McCallum J., Andrew N. (2021). Professional identity in nursing: A mixed method research study. Nurse Educ. Pract..

[29] Orr Z., Romem A. (2022). Holocaust and genocide studies in nursing education: Nurse practitioners as critical intellectuals. J. Nurs. Educ..

[30] Shields L., Hartin P., Shields K., Benedict S. (2015). Teaching the Holocaust in nursing and medical education in Australia. Work. Pap. Health Sci..

[31] Orr Z., Romem A. (2021). Teaching the holocaust in nursing schools: The perspective of the victims and survivors. Int. J. Environ. Res. Public Health.

[32] Czech H., Hildebrandt S., Reis S.P., Chelouche T., Fox M., González-López E., Lepicard E., Ley A., Offer M., Ohry P.A. (2023). The Lancet Commission on medicine, Nazism, and the Holocaust: Historical evidence, implications for today, teaching for tomorrow. Lancet.

[33] Ghetto Fighters’ House. The House. https://www.youtube.com/watch?v=pOEceBs-Jxs.

[34] Stanton G.H. The Ten Stages of Genocide. Genocide Watch.

[35] Kirkpatrick D.L., Brown S.M., Seidner C.J. (1998). The Four Levels of Evaluation. Evaluating Corporate Training: Models and Issues.

[36] Elo S., Kyngäs H. (2008). The qualitative content analysis process. J. Adv. Nurs..

[37] International Council of Nurses (2021). The ICN Code of Ethics for Nurses. https://www.icn.ch/sites/default/files/2023-06/ICN_Code-of-Ethics_EN_Web.pdf.

[38] Alanazi M.A., Shaban M.M., Ramadan O.M.E., Zaky M.E., Mohammed H.H., Amer F.G.M., Shaban M. (2024). Navigating end-of-life decision-making in nursing: A systematic review of ethical challenges and palliative care practices. BMC Nurs..

[39] Hardie P., Darley A., Langan L., Lafferty A., Jarvis S., Redmond C. (2022). Interpersonal and communication skills development in general nursing preceptorship education and training programs: A scoping review. Nurse Educ. Pract..

[40] Hart P.L., Brannan J.D., De Chesnay M. (2014). Resilience in nurses: An integrative review. J. Nurs. Manag..

[41] Orr Z., Machikawa E., Unger S., Romem A. (2022). Enhancing the structural competency of nurses through standardized patient simulation. Clin. Simul. Nurs..

[42] Orr Z., Zalcman B.G., Pinchas-Mizrachi R., Romem A. (2025). Fostering healthcare innovation: A mixed-methods study of an impact entrepreneurship course for nurse practitioner students. Nurs. Rep..

[43] Shadi A.Z., Zohreh V., Eesa M., Anoshirvan K. (2024). Moral sensitivity of nursing students: A systematic review. BMC Nurs..

[44] Shi X., Wang R., Li Y., Zeng L. (2025). A systematic review of educational interventions to enhance ethical sensitivity in nursing students in Asia and the Middle East. BMC Med. Ethics.

[45] Forte K., Higgins M., Pentz R.D. (2025). Fostering moral resilience: Evaluating a high-fidelity ethics simulation with prelicensure nursing students in their practice as new graduates. Nurse Educ..

[46] Venet A.S. (2024). Equity-Centered Trauma-Informed Education.

[47] Major R., Jackson C., Wareham J., Pidcock J. (2024). Supporting neurodivergent nursing students in their practice placements. Nurs. Stand..

[48] Cummings J., Serembus J.F., Ossont M.W. (2026). Supporting Neurodiversity: Effective Teaching Strategies for Nursing Students. Nurse Educ..

[49] Gonzalez L. (2024). Nursing education in the era of ChatGPT: Implications and opportunities. Online J. Issues Nurs..

[50] El Arab R.A., Al Moosa O.A., Abuadas F.H., Somerville J. (2025). The role of AI in nursing education and practice: Umbrella review. J. Med. Internet Res..

[51] Ma J., Wen J., Qiu Y., Wang Y., Xiao Q., Liu T., Zhang D., Zhao Y., Lu Z., Sun Z. (2025). The role of artificial intelligence in shaping nursing education: A comprehensive systematic review. Nurse Educ. Pract..

